# Hyperkalemia During Prolonged Anesthesia in a Greyhound

**DOI:** 10.1155/2024/3908979

**Published:** 2024-11-07

**Authors:** A. K. O'Neill

**Affiliations:** Anaesthesia Department, Moorong Veterinary Clinic, Wagga Wagga, New South Wales, Australia

**Keywords:** anesthesia, greyhound, hyperkalemia

## Abstract

**Case Report:** A 3-year-old female neutered greyhound presented for a dental procedure under general anesthesia. At the time of presentation, the dog was clinically well, with no health concerns from the client except for halitosis. The dog underwent general anesthesia with 13 teeth extracted and was stable until a severe, acute bradycardia was noticed at 2 h and 20 min postinduction. Venous blood analysis revealed a marked hyperkalemia. The dog was treated with calcium gluconate, an intravenous fluid bolus, glucose, and atropine. Serum potassium levels returned to within normal reference range at 60 min posttreatment. The dog developed ventricular tachycardia in recovery which responded to two lignocaine boluses. The dog was discharged from hospital in a stable condition.

**Conclusions:** Unanticipated hyperkalemia during anesthesia was treated in an otherwise healthy greyhound undergoing a dental procedure.

## 1. Introduction

Potassium is a tightly regulated cation present only in small quantities in extracellular fluid (2%), compared to intracellularly (98%), and is responsible for maintaining cell resting membrane potentional [1, 2]. Hyperkalemia is an increase in extracellular potassium and can be life-threatening in veterinary patients if not treated quickly, with excessive potassium reducing the resting membrane potential in cardiac cells, leading to an inactivation of voltage-dependent sodium channels and subsequently decreased conduction, severe arrythmias, and cardiac arrest [2]. Hyperkalemia-induced arrythmias are often seen in experimentally induced hyperkalemia, but not always in clinical/spontaneous scenarios [3]. When arrythmias are observed, peaked *T* waves, prolonged PR intervals, and eventually the disappearance of *P* waves are seen [3]. It has been reported that bradycardia secondary to hyperkalemia is minimally responsive to atropine due to this type of bradycardia not being vagally mediated [2].

Serum potassium may be increased due to increased intake, decreased urinary excretion, or intracellular to extracellular shifts [4]. Increased intake of potassium may be seen with potassium-containing intravenous fluid administration or oral potassium supplementation. Decreased urinary excretion of potassium can occur via several pathways. Potassium is secreted into the distal tubules of the kidney primarily due to concentration and electrochemical gradients and secreted into distal and collecting tubules via the increase in Na-K ATPase activity under the influence of aldosterone [5] (normally, 90%–95% of potassium is excreted renally, with the remainder passively secreted by the small intestine or actively by the colon) [6]. Kidney impairment (acute/chronic kidney disease, administration of potassium-sparing diuretics or nonsteroidal anti-inflammatories, or urinary obstruction) or hypoaldosteronism reduces the ability of the kidney to excrete potassium and leads to hyperkalemia [1]. The patient in this case received isotonic crystalloid fluids (Hartmann's) and did not have any clinical history or signs of renal disease or injury, although a complete renal profile was not performed. A shift of potassium extracellularly may be seen following administration of B-blockers or in cases of rhabdomyolysis, hyperthermia, or metabolic acidosis [7]. The venous blood gas results in this case reflect a respiratory alkalosis which is unlikely to have had an effect on serum potassium levels, although the blood collected was after isoflurane was ceased and the patient was hyperventilating, so the hyperkalemia may be explained by the anesthetic-induced respiratory depression which may lead to an efflux of potassium extracellularly [8]. The dog in this case also maintained a rectal temperature of 38.5°C or below so hyperthermia is also an unlikely cause.

Unanticipated acute hyperkalemia under anesthesia has been reported in the veterinary literature [9–13], although the etiology is largely unknown. These reports are most commonly seen in cases of prolonged anesthesia with the use of an alpha-2 agonists and in large feline species (tiger, lion, cheetah, and leopards) [14, 15]. In domestic species, it appears that greyhounds are more predisposed, although it is difficult to ascertain if this is a true predisposition or if identification of this phenomenon is biased towards greyhounds due to previous literature [12, 13]. There are occasional case reports of other breeds of dogs [10, 16].Although the etiology is not yet understood, prolonged duration of anesthesia is evident in many cases [9, 17]. It is important to note that anesthesia of greyhounds is safe, with hyperkalemia being an uncommon side effect. Knowledge of potential side effects and appropriate management, including adequately monitored recovery periods, is crucial.

Hyperkalemia during anesthesia has been reported in human medicine, with one study suggesting that it may be induced by propofol [18]. It is difficult to relate canine acute hyperkalemia under general anesthesia to propofol, as the majority of cases occur > 90 min after induction [17].

This case provides additional information on acute hyperkalemia of dogs under general anesthesia, particularly one that has not received an alpha-2 agonist, which is used in many case reports of hyperkalemia under general anesthesia in both dogs and nondomesticated species [9, 11, 14, 16].

## 2. Case Presentation

### 2.1. Case History

A 3-year-old female neutered greyhound presented for a dental procedure under general anesthesia. The owner of the dog reported halitosis at a dental consult 2 weeks prior and noted that food would get lodged between the dog's incisors, although the dog was otherwise well, eating, drinking, and toileting as normal. The dog was an ex-racer and was placed into a pet home with no known injuries or medical concerns.

On presentation, a thorough clinical examination was performed, with a heart rate of 140 bpm, strong synchronous pulses, pink mucous membranes, a capillary refill time of less than 1 s, and panting. Thoracic auscultation was normal with no heart murmur or abnormal lung sounds. Abdominal palpation was comfortable with no abnormalities noted. Rectal temperature was 38.5°C. The dog was very anxious in clinic.

A premedication of methadone (0.3 mg/kg) and acepromazine (0.02 mg/kg) was administered subcutaneously, and the dog was initiated on a balanced isotonic crystalloid (Hartmann's) at 3 mL/kg/h via a left cephalic catheter.

General anesthesia was induced with intravenous propofol (10 mg/mL), with a total of 14 mL administered, and intravenous fluids were increased to 5 mL/kg/h intraoperatively. The dog was intubated using a cuffed 12-mm endotracheal tube. Anesthesia was maintained with isoflurane, vaporized with 100% oxygen, and delivered via a circle rebreathing system. The dog breathed spontaneously for the entire anesthetic.

Blood pressure, SpO_2_, heart rate, respiratory rate, mucous membrane color, capillary refill time, and rectal temperature were monitored during anesthesia.

The heart rate varied from 60 to 115 bpm, the mean blood pressure varied from 74 to 100 mmHg, and the temperature varied from 38.8°C to 37.5°C while dental radiographs were performed. All teeth were probed and ultrasonically scaled and polished. Mandibular and maxillary dental nerve blocks were performed using a total of 1-mL lignocaine (20 mg/mL).

A total of 13 teeth were extracted based on radiographic evidence and clinical necessity, with the predominant reason being severe gingival recession. During tooth extraction at 60 min postinduction, tranexamic acid 10 mg/kg intravenously was administered due to haemorrhage from extraction sites.

### 2.2. Clinical Findings

Two hours and 20 minutes postinduction of anesthesia, a sudden and marked reduction in heart rate was observed, to 42 bpm.

### 2.3. Diagnostic Procedures

Once the bradycardia was identified, ECG leads were placed with a normal sinus rhythm present (Mindray multiparameter monitor, Mindray Medical, Notting Hill, New South Wales, Australia).

A venous blood sample was obtained from the right cephalic vein for electrolyte analysis (Catalyst One Chemistry Analyzer, IDEXX, Rydalmere, New South Wales, Australia) (*T* = 0 min). The result revealed a marked hyperkalemia (9.5 mmol/L, reference range 3.5–5.8 mmol/L) with normal sodium and chloride levels (Table 1). Blood glucose levels were 6.4 mmol/L (Alpha Trak 2, Zoetis, Kalamazoo, Michigan, United States).

### 2.4. Differential Diagnoses

• Decrease renal excretion of potassium
• Iatrogenic increased potassium load
• Transcellular potassium shift

In this case, the clients declined preanesthetic bloods so the lack of renal impairment, acidemia, rhabdomyolysis, hypoadrenocorticism, and insulin deficiency cannot be ruled out with certainty, although preoperative clinical examination and intraoperative bloodwork revealed no evidence of systemic disease. Nevertheless, the absence of clinical signs makes these differential diagnoses unlikely.

### 2.5. Treatment

Isoflurane was ceased. The final extraction pocket was being sutured when the bradycardia was noted so there was no need to continue anesthesia. The heart rate reduced further to 32 bpm while waiting for electrolyte results, and atropine 1 mL (600 *μ*g/mL) was administered intravenously.

Once the hyperkalemia was identified, 10% calcium gluconate (0.5 mL/kg) was administered intravenously over 5 min. An isotonic crystalloid fluid bolus of 10 mL/kg (Hartmann's) was administered intravenously over 10 min with the idea of diluting serum potassium. Fifty percent of glucose (0.5 mL/kg) diluted 1:1 with isotonic crystalloid was administered intravenously over 10 min.

Repeat venous blood analysis (Coag Dx Analyzer; IDEXX, Rydalmere, New South Wales, Australia) showed a reduction of serum potassium to 9.3 mmol/L at *T* = 30 min.

The dogs heart remained below 50 bpm so an additional 0.6-mL atropine was administered intravenously, to bring the total dose to 1.6 mL (600 *μ*g/mL).

The heart rate slowly increased to 110 bpm at 45 min posttreatment with calcium gluconate and glucose. Initial recovery from general anesthetic was uneventful, and ECG monitoring was continued into recovery with a normal sinus rhythm and a heart rate of 120 to 150 bpm.

Repeat venous blood gas sample obtained at *T* = 60 min showed significant reduction in serum potassium (5.5 mmol/L) within normal reference range. Venous blood gas results also revealed a respiratory alkalosis (pH 7.54, PCO_2_ 28 mmHg, and PO_2_ 147 mmHg), likely explained by the dog hyperventilating in recovery.

Repeat venous blood gas sample obtained at *T* = 120 min showed a further decrease in serum potassium (3.5 mmol/L) within normal reference range. Chloride increased further (although still a mild elevation) to 115 mmol/L. Venous blood gas sample continued to reflect a respiratory alkalosis (pH 7.44, PCO_2_ 34 mmHg, and PO_2_ 92 mmHg).

Four hours and 40 minutes postinduction of anesthesia, and in recovery, the heart rate suddenly elevated to 190 bpm. The dog was panting and anxious when moved or touched.

Buprenorphine 1.6 mL IV (0.3 mg/mL) was administered for pain relief in an attempt to reduce the tachycardia although no response was seen. The ECG trace remained normal, and the systolic blood pressure varied from 120 to 150 mmHg. The temperature varied from 38.1°C to 38.2°C postoperatively.

The dog remained tachycardic but stable until 5 h and 50 min postinduction, where ventricular tachycardia was noted. Lignocaine 3 mL (20 mg/mL) was administered intravenously over 1 min and resulted in temporary resolution of the ventricular tachycardia, although fell back into ventricular tachycardia 4 min following the lignocaine bolus. An additional 3-mL lignocaine (20 mg/mL) was administered intravenously which resolved the ventricular tachycardia.

Gabapentin 800 mg was administered orally in an attempt to reduce the patient's anxiety.

### 2.6. Outcome

The dog was monitored in hospital for another 3 h and then discharged. The ECG trace and blood pressure remained stable and normal until discharge.

The dog was sent home on meloxicam 0.1 mg/kg for 5 days.

Two weeks postdischarged, the patient returned for a recheck where the owners reported the dog had been doing well at home, with no concerns. Repeat serum potassium concentration was normal, and the ECG trace showed normal sinus rhythm.

## 3. Discussion

This case describes an acute hyperkalemic event in a 3-year-old female neutered greyhound, detected by an acute and severe bradycardia at 2 h and 20 min into propofol-induced anesthesia. There were no abnormalities detected on ECG, and the bradycardia was unresponsive to anticholinergic treatment. Electrolyte analysis and blood gases revealed a marked hyperkalemia and mild alkalosis, although blood gases were not performed until after isoflurane was ceased and the dog was hyperventilating. The dog developed lignocaine-responsive ventricular tachycardia in recovery and was discharged from hospital in a stable condition.

The etiology of anesthesia-induced hyperkalemia is unknown in domestic dogs although there a retrospective study of acute hyperkalemia under anesthesia in dogs revealed that all dogs in the study were under general anesthesia for > 60 min [17]. This aligns with the current case report where anesthesia was prolonged and with other similar studies [9].

This case report aligns with others in terms of prolonged anesthesia, but it does not support the possibility of alpha-2 agonists being the underlying cause. It is thought that alpha-2 agonists may cause a hyperglycemia resulting in a hyperkalemia, by inhibiting the release of insulin from pancreatic beta cells [14, 15]. The majority of case reports available to the author suggests that alpha-2 agonists usually administered as a premedication may play a role in anesthesia-induced hyperkalemia [9, 14, 16, 17]. No alpha-2 agonists were used in this case, and hyperglycemia was not present, which provides important information to add to the literature in terms of possible etiologies. Further case reports are required without the inclusion of medications like medetomidine, to truly reflect possible causes of anesthesia-induced hyperkalemia.

High levels of serum potassium are responsible for depression of cardiac myocyte excitability and conduction of action potentials, resulting in a bradycardia that is often nonresponsive to anticholinergic treatment [2], as seen in this case. There are cases of hyperkalemia-induced bradycardia reported where anticholinergic treatment has been successful, with the initiation of treatment with calcium gluconate suspected to play a role [9]. In this case, it was important to note the marked tachycardia and subsequent ventricular tachycardia observed once serum potassium levels returned to normal. Atropine was administered up until 30 min posthyperkalemia identification due to severe and declining bradycardia, with serum potassium levels returning to normal 30 min after the last atropine administration. This means it is possible that atropine was still exerting effects at the time where potassium returned to normal (atropine may have effects against bradycardia for 50 min) [19] and may have induced the tachycardia).

Calcium gluconate and glucose were used to treat the hyperkalemia-induced bradycardia in this case. Calcium is known to antagonize the effects of potassium on the cell membrane and restore the resting membrane potential in cardiac myocytes which increases excitability [20]. These affects only last for up to 60 min and does not reduce serum potassium levels, so a definitive treatment is required for the hyperkalemia [20]. The aim of treatment in this case was to encourage potassium influx into cells, achieved with administration of glucose which increases endogenous insulin and drives potassium into cells. Other studies have suggested the use of insulin, in combination with glucose to push potassium from the extracellular space, intracellularly [1, 12, 20]. Insulin was not used in this case due to the concern of causing subsequent hypoglycemia, although in future cases may show a quicker return of normokalaemia and a normal heart rate compared to the current case.

The greyhound in this case report was normal on clinical examination prior to general anesthesia, with no evidence of systemic disease. Given the lack of history for renal dysfunction in a young, clinically well greyhound, it is unlikely that renal disease played a role in the hyperkalemia under general anesthesia. Ideally, preanesthetic bloods would have been undertaken to rule out significant renal dysfunction, although in this case was declined by the client. Similarly, it has been shown that endocrinopathies such as hypoadrenocorticism or diabetes may cause hyperkalemia in dogs, and even though no clinical or historical evidence for either of these diseases was present in this case, the lack of preanesthetic bloods cannot rule them out entirely. The intraoperative glucose was 6.4 mmol/L, and the serum sodium level was within normal limits in all electrolyte and blood gas analysis which makes endocrinopathies unlikely in this case. It would be recommended to complete full blood analysis prior to all cases of prolonged anesthesia to better understand preanesthetic indictors of hyperkalemia, with the aim to prevent life-threatening bradycardia, although when compared to other studies, it is difficult to predict whether such screening tests would provide any further information.

In summary, acute bradycardia caused by hyperkalemia during general anesthesia is a potentially life-threatening occurrence seen in both domestic and nondomestic veterinary species. In this case, the dog was discharged with no further clinical signs after treatment with calcium gluconate and glucose, although there is a need for further reporting to better understand the etiology of this process to help prevent rather than treat it.

## Figures and Tables

**Table 1:** Venous blood gas and electrolyte analysis following identification of hyperkalemia at *T* = 0 min.

	**Time**			
**Parameter**	**T** = 0 min	**T** = 30 min	**T** = 60 min	**T** = 120 min
K^+^ (3.5–5.8 mmol/L)	9.5	9.3	5.5	3.5
Na^+^ (144–160 mmol/L)	149	146	152	155
Cl^−^ (109–122 mmol/L)	111	109	113	115
Blood glucose (mmol/L)	6.4	9.3	7.1	

## Data Availability

The data that support the findings of this study are available from the corresponding author upon a reasonable request.

## Nomenclature

°C: degrees Celsius
bpm: beats per minute
ECG: electrocardiogram
h: hour
kg: kilogram
mL: milliliter
mmHg: millimeters of mercury
mmol/L: millimole per liter
